# Knowledge and Opinions of Healthcare Professionals about Thirdhand Smoke: A Multi-National, Cross-Sectional Study

**DOI:** 10.3390/healthcare10050945

**Published:** 2022-05-19

**Authors:** Blanca Quispe-Cristóbal, Cristina Lidón-Moyano, Juan Carlos Martín-Sánchez, Hipólito Pérez-Martín, Àurea Cartanyà-Hueso, Íñigo Cabriada-Sáez, Sonia de Paz-Cantos, Jose M. Martínez-Sánchez, Adrián González-Marrón

**Affiliations:** 1 Group of Evaluation of Health Determinants and Health Policies, Department of Basic Sciences, Universitat Internacional de Catalunya, 08195 Sant Cugat del Vallès, Spain; bsquispe@uic.es (B.Q.-C.); clidon@uic.es (C.L.-M.); jcmartin@uic.es (J.C.M.-S.); hperez@uic.es (H.P.-M.); cartanya@uji.es (À.C.-H.); icabriada@uic.es (Í.C.-S.); sdepaz@uic.es (S.d.P.-C.); jmmartinez@uic.es (J.M.M.-S.); 2 Group of Perinatal Epidemiology, Environmental Health, and Clinical Research, Department of Medicine, School of Health Sciences, Universitat Jaume I, Av. Vicent Sos Baynat, s/n, 12071 Castelló de la Plana, Spain

**Keywords:** health knowledge, attitudes, practice, third-hand smoke, health personnel

## Abstract

There is scarce evidence on the knowledge and opinions about third-hand smoke (THS) of health care professionals. The main aim of this study was to explore the knowledge and opinions of health care professionals about THS and, secondarily, to explore the factors that are associated with this knowledge. Cross-sectional study using a snowball sample of multi-national health care professionals (n = 233). Data were obtained from an exploratory, online questionnaire. The health care professionals’ knowledge and opinions on THS were described with absolute frequency and percentage. Chi-square and Fisher-Freeman-Halton exact tests, and simple logistic regression models, were used to explore the bivariate association between the knowledge of the concept THS and sex, continent of birth, educational level, occupation, years of experience, and attitude towards smoking. Finally, a multivariable logistic regression model incorporating all the above variables was fitted. A total of 65.2% of the participants were unaware of the term THS before the study began. In the bivariate analysis, an association was found between prior knowledge of the term THS and continent of birth (*p*-value = 0.030) and occupation (*p*-value = 0.014). In the multivariable logistic regression model, a significant association was observed between prior knowledge of the concept THS and sex (*p*-value = 0.005), continent of birth (*p*-value = 0.012), and occupation (*p*-value = 0.001). Almost two out of three health care professionals who participated in our study did not know what THS was. Educational activities on this topic should be implemented.

## 1. Introduction

There is a direct relationship between tobacco use and premature morbidity and mortality in the smoking and non-smoking population [1]. According to the World Health Organization (WHO), more than 8 million people die each year from tobacco use worldwide [2]. Of these, about 7 million are active smokers and 1.2 million passive smokers. Of the latter, 65,000 are children under 5 years of age who die from diseases attributable to exposure to secondhand smoke (SHS) [2].

SHS is the smoke exhaled by the smoker and the sidestream smoke generated from the burning tip of the tobacco product [3]. SHS contains thousands of harmful chemicals. Among these substances are benzene, cadmium, arsenic, or nickel, which the International Agency for Research on Cancer includes in the Group 1 of human carcinogens [4]. Exposure to SHS is a risk factor for cardiovascular and respiratory diseases in adults [5] and also impacts children’s health [6].

Passive exposure to tobacco smoke includes not only exposure to SHS, for which there is ample evidence of its harmful effects, but also to thirdhand smoke (THS). THS refers to the secondary pollutants and by-products of tobacco smoke that persist in the environment after a tobacco product is extinguished [7]. These products include, among others, semivolatile and nonvolatile oxidized and nitrosated compounds and volatile compounds, such as furans or nitriles [8]. Exposure to THS occurs when the passively exposed person inhales, ingests or absorbs through the skin the by-products that remain on hard surfaces such as furniture, clothing, walls, and even hair [7]. In this regard, it has been shown that the homes of former smokers are contaminated by THS up to 6 months after they have quit smoking [9]. Although the effect on health of THS exposure should be still fully assessed, THS exposure has been preliminarily associated with detrimental health disorders (e.g., aggregation of platelets in the offspring [10], asthma [11], reproductive system conditions [12]).

The tobacco epidemic is a major public health problem that needs to be addressed from different sectors and levels. In this regard, health professionals, especially those in the health care area, can play a fundamental role since they have direct contact with the population and influence their behaviors, and can therefore impact on the patients’ health outcomes [13]. For this reason, and due to the scarce information currently available on THS, it is essential to determine the knowledge and opinions of health care professionals on THS, as well as to explore the factors associated with this knowledge, since their advice could help to promote people’s health and thus reduce the health impact associated with THS exposure [14].

Therefore, the aim of this study was to describe the knowledge and opinions of health care professionals about THS and explore what factors are associated with prior knowledge of the concept THS.

## 2. Materials and Methods

### 2.1. Design

Cross-sectional study using a snowball sample recruited from April to June 2021.

### 2.2. Population and Sample

Practicing healthcare professionals from Africa, America, Asia, and Europe (n = 233). The link to the questionnaire was shared with first and last authors’ professional contacts from different countries, working in clinics and public and private hospitals, who were asked to share the link with their colleagues. Inclusion criteria were having intermediate or higher education in Health Sciences (e.g., Nursery, Medicine), having healthcare functions at the time of completing the questionnaire and giving prior consent to participate in the study.

### 2.3. Instrument

Online, ad-hoc questionnaire implemented in Google Forms in English, Spanish, and German. Some of the questions were retrieved from questionnaires used in two previous studies, one on the knowledge about THS in health care professionals in the US [15] and another on the knowledge about THS in parents of children under 3 years of age in Spain [16].

The questionnaire was divided into two parts: the first part addressed questions on the knowledge and opinions of the participants on THS and the second part included sociodemographic questions, related to their healthcare activity and current attitude towards smoking.

Before starting the questionnaire, the participants had access to the information sheet and the informed consent form, which they were required to fill in before answering the questionnaire.

### 2.4. Variables

Prior knowledge of the term THS was collected through the question “Have you ever heard of THS?” (“yes”, “no”, “don’t know/no answer”). Opinions on the knowledge that other people had about THS were collected through the questions “How informed do you think your coworkers in the health care setting are on THS?” and “How informed do you think the general population is about THS?”, both with the possible answers “not at all informed”, “somewhat informed”, “very informed”, and “don’t know/no answer”. Additionally, “Parents are knowledgeable about THS”, with the possible options “strongly disagree”, “disagree”, “neither agree/nor disagree”, “agree”, “strongly agree”, “don’t know/don’t answer”.

Own opinions on THS and the consequences of its exposure were obtained through the questions “How much attention do you think THS is receiving in the health care setting?”, with four possible answers: “not enough attention”, “just the right amount of attention”, “a lot of attention”, “don’t know/no answer”; “how much do you think THS is harmful to health?” and “how much do you think THS affects children?” with five possible answers: “not at all”, “a little”, “quite a lot”, “a lot”, “don’t know/no answer”.

Finally, beliefs of health care professionals on the recommendations health professionals should provide to the general population regarding THS were assessed through the questions “Health care professionals should promote habits of hand hygiene, mouth hygiene, etc. among smokers with children under 3 years of age to prevent exposure to THS among the child population” and “Health professionals should inform about the effects of passive smoking exposure (SHS and THS) to parents of children under 3 years of age”, with six possible answers: “strongly disagree”, “disagree”, “neither agree/disagree”, “agree”, “strongly agree”, “do not know/do not answer”.

### 2.5. Covariates

Respondents were asked to indicate their sex (male, female), age, country of birth, level of education (non-university, diploma/graduate/graduate, master’s, or doctorate), current occupation (doctor, nurse, or open ended answer, later grouped as “other”), professional experience in years (less than 1, between 1 and 5, between 6 and 10, between 11 and 15, and more than 15), and current smoking behavior (never smoker, former smoker, or current smoker).

### 2.6. Statistical Analysis

Categorical variables were described with absolute frequency and percentage and age with mean and standard deviation. To explore the association between previous knowledge of the term THS and each of the covariates, Chi-square and Fisher-Freeman-Halton exact tests were carried out, and simple logistic regression models were applied to estimate odds ratios (OR) and their 95% confidence intervals (95% CI). To estimate the adjusted ORs (aOR) and their 95% CIs, a multivariable logistic regression model was performed.

The significance level was set at 0.05 and the contrasts were two-sided. The statistical program used was IBM SPSS v.26.

## 3. Results

The final sample consisted of 233 participants born in 24 countries. A total of 83.7% were female; 61.0% had a bachelor’s, undergraduate, or degree; 67.4% were nurses; 52.4% had more than 15 years of experience; and 71.0% were nonsmokers (Table 1).

Before introducing the definition of THS, 65.2% of the participants had not heard of this term (Table 2). Once it was explained, 74.2% of the participants considered that THS was at least quite harmful to health and 89.3% at least quite harmful to children. However, 76.7% believed that THS is not receiving enough attention in the health care setting.

In the bivariate analysis, prior knowledge of the THS concept was significantly associated with continent of birth (*p*-value = 0.030), occupation (*p*-value = 0.014), and with prior knowledge of the concept SHS (*p*-value < 0.001) (Table 3).

In the multivariable logistic regression model, a significant association was obtained between prior knowledge of the THS concept and sex (*p*-value = 0.005), continent of birth (*p*-value = 0.012), and occupation (*p*-value = 0.001) (Table 4).

## 4. Discussion

Almost two out of three healthcare professionals in the healthcare area were unaware of the term THS at the beginning of the study. This lack of knowledge was associated with male sex, continent of birth, and a current occupation other than physician. Once the concept of THS was presented, about three out of four participants considered THS to be at least quite harmful to health and about nine out of ten participants at least quite harmful to children.

To our knowledge, the exploration of the knowledge and opinions on THS of health personnel had only been carried out at a single-center level in the US [15]. Therefore, this would be the first study to describe the knowledge and opinions on THS in health professionals in the health care area at a multinational level. Importantly, we have obtained a similar estimation of the prior knowledge of the concept THS (around one third) than in Darlow et al. [15]

It is worrying to point out that an important proportion of participants in our study, more than three out of four, estimated that in the health care setting where they work, THS is not receiving enough attention and that about 50% of their coworkers are not informed about THS. These considerations, associated with the evidence that is progressively accumulating on the harmful effects of exposure to THS [10,17], leave a great margin for improvement in terms of training on THS. In this sense, training health professionals in smoking cessation has proved effective in the reduction of prevalence of smoking and abstinence [18]. Hence, we propose incorporating educational activities on tobacco control both in educational centers (e.g., universities) and in healthcare settings, where such have not been implemented thus far.

The vast majority of the participants (over 80%) agreed or strongly agreed that health professionals should promote hygiene habits for smokers with children under 3 years of age in order to prevent exposure to THS among the child population, as well as to inform parents of children under 3 years of age (over 90%) about the effects of passive exposure to tobacco. From this, it can be inferred that health care personnel themselves consider that they should play an active role in the empowerment and health education of the people with whom they come into contact with regard to this exposure. This is in line with Article 14 of the WHO Framework Convention on Tobacco Control, which states that health workers should participate in the diagnosis and treatment of tobacco dependence and counselling services on cessation of tobacco use [19]. In our opinion, we consider that it would be especially interesting to involve healthcare personnel from the pediatric area because of their close contact with children, one of the groups most vulnerable to exposure to THS.

### Limitations

The main limitation of our study is based on the fact that we have used a data collection instrument that has not undergone a process of cultural adaptation and validation. This has occurred, fundamentally, due to the fact that THS is a relatively new field of research. In fact, scales to assess THS knowledge, attitudes, and behaviors are currently being developed and validated [20]. However, we adapted some items used in other questionnaires from published studies, such as those from a questionnaire used in health professionals carried out in the US [15] and those from another questionnaire on knowledge and attitudes about THS among parents with children under 3 years of age in Spain [16]. On the other hand, and associated with the fact of collecting information through a questionnaire, there is a risk of self-reporting bias. In addition, the snowball sampling carried out and the sensitive selection criteria used, fundamentally exploratory, prevent conclusions from being drawn about specific populations, which should be better defined in future studies. In this sense, and associated to a potential volunteer bias (i.e., professionals who have participated in this research may be the ones more aware of the concept THS, and more willing to participate) we believe that the proportion of professionals knowledgeable of the concept THS may be even lower. Finally, it is likely that incorporating a qualitative approach would have allowed a deeper exploration of the opinions of health care professionals on THS. In this sense, it would be interesting to carry out mixed-methods studies in the future that would allow incorporating this methodology.

## 5. Conclusions

Almost two out of three health care professionals who participated in our study did not know what the concept THS was before starting our study. Educational activities to raise awareness on this topic should be implemented.

## Figures and Tables

**Table 1:** Sample characteristics (n = 233).

Sex	
Female	190 (83.7)
Male	37 (16.3)
**Age [x^−^ (SD)]**	45.2 (11.1)
**Continent of Birth**	
Africa	2 (0.9)
America	93 (40.1)
Asia	3 (1.3)
Europe	129 (56.8)
**Educational Level**	
Non-university	8 (3.5)
Bachelor’s, undergraduate, degree	139 (61.0)
Master’s	68 (29.8)
Doctorate	13 (5.7)
**Occupation**	
Nurse	153 (67.4)
Physician	43 (18.9)
Other	31 (13.7)
**Years of Experience**	
Less than 1	6 (2.6)
Between 1 and 5	29 (12.4)
Between 6 and 10	36 (15.5)
Between 11 and 15	38 (16.3)
Over 15	122 (52.4)
**Attitude Towards Smoking**	
Never smoker	164 (71.0)
Former smoker	48 (20.8)
Current smoker	19 (8.2)

Results expressed as absolute frequency and %, unless otherwise expressed. % of total valid cases for each variable. x^−^: arithmetic mean. SD: standard deviation.

**Table 2:** Response to questions on prior knowledge and opinions about third hand smoke.

	n (%)
**Have you ever heard of third-hand smoke?**	
Yes	81 (34.8)
No	152 (65.2)
Don’t know/no answer	0 (0.0)
**How much attention do you think third-hand smoke is receiving in the health care setting?**	
Not enough attention	178 (76.7)
Just the right amount of attention	15 (6.5)
A lot of attention	7 (3.0)
Don’t know/no answer	32 (13.8)
**How informed do you think your colleagues in the care setting are about third-hand smoke?**	
Not informed at all	103 (44.2)
Somewhat informed	107 (45.9)
Very informed	8 (3.5)
Don’t know/no answer	15 (6.4)
**How informed do you think the general population is about third-hand smoke?**	
Not informed at all	68 (29.2)
Somewhat informed	156 (67.0)
Very informed	0 (0.0)
Don’t know/no answer	9 (3.8)
**How much do you think third-hand smoke is harmful to health?**	
Not at all	8 (3.4)
A little	29 (12.5)
Quite a lot	83 (35.6)
A lot	90 (38.6)
Don’t know/no answer	23 (9.9)
**How much do you think third-hand smoke affects children?**	
Not at all	3 (1.3)
A little	11 (4.7)
Quite a lot	70 (30.1)
A lot	138 (59.2)
Don’t know/no answer	11 (4.7)
**Parents are knowledgeable about THS**	
Strongly disagree	46 (22.2)
Disagree	90 (43.5)
Neither agree nor disagree	23 (11.1)
Agree	17 (8.2)
Strongly agree	7 (3.4)
Don’t know/no answer	24 (11.6)
**Health professionals should promote hand, mouth, etc. hygiene habits among smokers with children under 3 years of age to prevent exposure to third-hand smoke among children**	
Strongly disagree	11 (4.7)
Disagree	11 (4.7)
Neither agree nor disagree	14 (6.0)
Agree	82 (35.2)
Strongly agree	114 (49.0)
Don’t know/no answer	1 (0.4)
**Health professionals should inform parents of children under 3 years of age about the effects of passive smoking (secondhand smoke and thirdhand smoke)**	
Strongly disagree	9 (4.0)
Disagree	4 (1.8)
Neither agree nor disagree	8 (3.5)
Agree	62 (26.7)
Strongly agree	147 (63.1)
Don’t know/no answer	2 (0.9)

n: absolute frequency %: percentage.

**Table 3:** Association between prior knowledge of the concept thirdhand smoke and covariates.

	Prior Knowledge of the Term Thirdhand Smoke		
	No	Yes	*p*-Value
**Sex**			0.115
Male	28 (75.7)	9 (24.3)	
Female	118 (62.1)	72 (37.9)	
**Continent of Birth**			0.030
Africa	2 (100.0)	0 (0.0)	
America	53 (57.0)	40 (43.0)	
Asia	1 (33.3)	2 (66.7)	
Europe	93 (72.1)	36 (27.9)	
**Educational Level**			0.190
Non-university	8 (100.0)	0 (0.0)	
Bachelor’s, undergraduate, degree	87 (62.6)	52 (37.4)	
Master’s	44 (64.7)	24 (35.3)	
Doctorate	9 (69.2)	4 (30.8)	
**Occupation**			0.014
Nurse	102 (66.7)	51 (33.3)	
Physician	21 (48.8)	22 (51.2)	
Other	25 (80.6)	6 (19.4)	
**Years of Experience**			0.651
Less than 1	5 (83.3)	1 (16.7)	
Between 1 and 5	21 (72.4)	8 (27.6)	
Between 6 and 10	23 (63.9)	13 (36.1)	
Between 11 and 15	26 (68.4)	12 (31.6)	
Over 15	75 (61.5)	47 (38.5)	
**Attitude Towards Smoking**			0.680
Never smoker	106 (64.4)	58 (35.4)	
Former smoker	30 (62.5)	18 (37.5)	
Current smoker	14 (73.7)	5 (26.3)	
**Knowledge of the Concept Secondhand Smoke**			0.001
Yes	81 (43.8)	104 (56.2)	
No	46 (100.0)	0 (0.0)	

Results expressed in absolute frequency and percentage. *p*-value obtained from Chi-square test of independence or Fisher-Freeman-Halton test.

**Table 4:** Results of the simple and multiple logistic regression models on the variable prior knowledge of the concept thirdhand smoke.

	Prior Knowledge of the Term Thirdhand Smoke			
	OR (95% CI)	*p*-Value	aOR (95% CI)	*p*-Value
**Sex**		0.119		0.005
Male	0.527 (0.235–1.180)		0.209 (0.070–0.622)	
Female	Reference		Reference	
**Continent of Birth**		0.087		0.012
Africa	NA		NA	
America	Reference		Reference	
Asia	2.650 (0.232–30.261)		2.116 (0.172–25.993)	
Europe	0.513 (0.292–0.900)		0.321 (0.157–0.655)	
**Educational Level**		0.965		0.735
Non-university	NA		NA	
Bachelor’s, undergraduate, degree	1.345 (0.394–4.587)		1.641 (0.394–6.841)	
Master’s	1.227 (0.342–4.407)		1.132 (0.263–4.878)	
Doctorate	Reference		Reference	
**Occupation**		0.017		0.001
Nurse	0.477 (0.240–0.948)		0.169 (0.064–0.445)	
Physician	Reference		Reference	
Other	0.229 (0.078–0.670)		0.131 (0.033–0.521)	
**Years of Experience**		0.666		0.433
Less than 1	0.319 (0.036–2.817)		0.218 (0.021–2.288)	
Between 1 and 5	0.608 (0.249–1.484)		0.461 (0.153–1.384)	
Between 6 and 10	0.902 (0.417–1.951)		0.590 (0.226–1.540)	
Between 11 and 15	0.736 (0.339–1.599)		0.693 (0.294–1.633)	
Over 15	Reference		Reference	
**Attitude Towards Smoking**		0.684		0.827
Never smoker	0.653 (0.224–1.903)		0.695 (0.199–2.429)	
Former smoker	1.097 (0.563–2.135)		0.797 (0.203–3.126)	
Current smoker	Reference		Reference	

OR: odds ratio. aOR: adjusted odds ratio. 95% CI: 95% confidence interval. NA: not applicable.

## Data Availability

Not applicable.

## References

[1] GBD 2015 Tobacco Collaborators (2017). Smoking prevalence and attributable disease burden in 195 countries and territories, 1990–2015: A systematic analysis from the Global Burden of Disease Study 2015. Lancet.

[2] Tobacco—Fact Sheet. https://www.who.int/news-room/fact-sheets/detail/tobacco.

[3] National Research Council (US) Committee on Passive Smoking (1986). Washington (DC) Environmental Tobacco Smoke: Measuring Exposures and Assessing Health Effects.

[4] Agents Classified by the IARC Monographs. Volume 1–130. https://monographs.iarc.who.int/agents-classified-by-the-iarc/.

[5] Office on Smoking and Health (US) (2006). Atlanta (GA) the Health Consequences of Involuntary Exposure to Tobacco Smoke: A Report of the Surgeon General. Cardiovascular Diseases from Exposure to Secondhand Smoke.

[6] Nadhiroh S.R., Djokosujono K., Utari D.M. (2020). The association between secondhand smoke exposure and growth outcomes of children: A systematic literature review. Tob. Induc. Dis..

[7] Matt G.E., Quintana P.J.E., Destaillats H., Gundel L.A., Sleiman M., Singer B.C., Jacob P., Benowitz N., Winickoff J.P., Rehan V. (2011). Thirdhand tobacco smoke: Emerging evidence and arguments for a multidisciplinary research agenda. Environ. Health Perspect..

[8] Jacob P., Benowitz N.L., Destaillats H., Gundel L., Hang B., Martins-Green M., Matt G.E., Quintana P.J.E., Samet J.M., Schick S.F. (2017). Thirdhand Smoke: New Evidence, Challenges, and Future Directions. Chem. Res. Toxicol..

[9] Matt G.E., Quintana P.J.E., Zakarian J.M., Hoh E., Hovell M.F., Mahabee-Gittens M., Watanabe K., Datuin K., Vue C., Chatfield D.A. (2016). When smokers quit: Exposure to nicotine and carcinogens persists from thirdhand smoke pollution. Tob. Control.

[10] Ali H.E.A., Alarabi A.B., Karim Z.A., Rodriguez V., Hernandez K.R., Lozano P.A., El-Halawany M.S., Alshbool F.Z., Khasawneh F.T. (2022). In utero thirdhand smoke exposure modulates platelet function in a sex-dependent manner. Haematologica.

[11] Yu M., Mukai K., Tsai M., Galli S.J. (2018). Thirdhand smoke component can exacerbate a mouse asthma model through mast cells. J. Allergy Clin. Immunol..

[12] Liu H., Chen H. (2022). The effects of thirdhand smoke on reproductive health. J. Appl. Toxicol..

[13] Chipidza F.E., Wallwork R.S., Stern T.A. (2015). Impact of the Doctor-Patient Relationship. Prim. Care Companion CNS Disord..

[14] Hang B., Wang P., Zhao Y., Sarker A., Chenna A., Xia Y., Snijders A.M., Mao J.-H. (2017). Adverse Health Effects of Thirdhand Smoke: From Cell to Animal Models. Int. J. Mol. Sci..

[15] Darlow S.D., Heckman C.J., Munshi T., Collins B.N. (2017). Thirdhand smoke beliefs and behaviors among healthcare professionals. Psychol. Health Med..

[16] Díez-Izquierdo A., Cassanello P., Cartanyà A., Matilla-Santander N., Balaguer Santamaria A., Martinez-Sanchez J.M. (2018). Knowledge and attitudes toward thirdhand smoke among parents with children under 3 years in Spain. Pediatr. Res..

[17] Kelley S.T., Liu W., Quintana P.J.E., Hoh E., Dodder N.G., Mahabee-Gittens E.M., Padilla S., Ogden S., Frenzel S., Sisk-Hackworth L. (2021). Altered microbiomes in thirdhand smoke-exposed children and their home environments. Pediatr. Res..

[18] Carson K.V., Verbiest M.E.A., Crone M.R., Brinn M.P., Esterman A.J., Assendelft W.J.J., Smith B.J. (2012). Training health professionals in smoking cessation. Cochrane Database Syst. Rev..

[19] WHO Framework Covention on Tobacco Control. https://apps.who.int/iris/rest/bitstreams/50793/retrieve.

[20] Record R.A., Greiner L.H., Wipfli H., Pugel J., Matt G.E. (2022). Thirdhand Smoke Knowledge, Attitudes, and Behavior: Development of Reliable and Valid Self-Report Measures. Nicotine Tob. Res..

